# Visual Function and Cortical Organization in Carriers of Blue Cone Monochromacy

**DOI:** 10.1371/journal.pone.0057956

**Published:** 2013-02-28

**Authors:** Ethan A. Rossi, Rebecca L. Achtman, Arnaud Guidon, David R. Williams, Austin Roorda, Daphne Bavelier, Joseph Carroll

**Affiliations:** 1 School of Optometry, University of California, Berkeley, Berkeley, California, United States of America; 2 Rochester Center for Brain Imaging, University of Rochester, Rochester, New York, United States of America; 3 Center for Visual Science, University of Rochester, Rochester, New York, United States of America; 4 Department of Brain and Cognitive Sciences, University of Rochester, Rochester, New York, United States of America; 5 Brain Imaging and Analysis Center, Duke University, Durham, North Carolina, United States of America; 6 Department of Ophthalmology, Medical College of Wisconsin, Milwaukee, Wisconsin, United States of America; 7 Department of Cell Biology, Neurobiology, and Anatomy, Medical College of Wisconsin, Milwaukee, Wisconsin, United States of America; 8 Department of Biophysics, Medical College of Wisconsin, Milwaukee, Wisconsin, United States of America; National Eye Institute, United States of America

## Abstract

Carriers of blue cone monochromacy have fewer cone photoreceptors than normal. Here we examine how this disruption at the level of the retina affects visual function and cortical organization in these individuals. Visual resolution and contrast sensitivity was measured at the preferred retinal locus of fixation and visual resolution was tested at two eccentric locations (2.5° and 8°) with spectacle correction only. Adaptive optics corrected resolution acuity and cone spacing were simultaneously measured at several locations within the central fovea with adaptive optics scanning laser ophthalmoscopy (AOSLO). Fixation stability was assessed by extracting eye motion data from AOSLO videos. Retinotopic mapping using fMRI was carried out to estimate the area of early cortical regions, including that of the foveal confluence. Without adaptive optics correction, BCM carriers appeared to have normal visual function, with normal contrast sensitivity and visual resolution, but with AO-correction, visual resolution was significantly worse than normal. This resolution deficit is not explained by cone loss alone and is suggestive of an associated loss of retinal ganglion cells. However, despite evidence suggesting a reduction in the number of retinal ganglion cells, retinotopic mapping showed no reduction in the cortical area of the foveal confluence. These results suggest that ganglion cell density may not govern the foveal overrepresentation in the cortex. We propose that it is not the number of afferents, but rather the content of the information relayed to the cortex from the retina across the visual field that governs cortical magnification, as under normal viewing conditions this information is similar in both BCM carriers and normal controls.

## Introduction

The normal human visual system is extremely variable in both structure and function. Peak cone density varies over a factor of 3 in normal eyes [1], with the ratio of long- (L-) to middle- (M-) wavelength sensitive cones varying from between 1∶1 to 17∶1 in persons with normal color vision [2], [3]. This variability extends to subcortical areas, with lateral geniculate nucleus (LGN) volume showing a 2–3 fold range [4], [5], and to the cortex, with the surface area of primary visual cortex (V1) varying by as much as a factor of three [6], [7]. Visual function is also highly variable, as best corrected visual acuity (VA) ranges over a factor of 2 in normally sighted individuals [8].

Coordinated size variation has been observed between the different visual system components within an individual [9] and this has led to the suggestion that a single factor may determine the dimensions of the visual system. Several studies have supported this view by establishing that the development of different visual components are indeed interdependent [6], [9]–[11]. It has been hypothesized that the cone mosaic may be the key factor that leads to the variability observed in higher structures [6], [12]. In this model, it is hypothesized that cone density governs retinal ganglion cell (RGC) density, which in turn determines the retinotopic organization of the cortex. This is consistent with a model put forth by Wässle et al., who stated that ganglion cell density drives the retinotopic organization of the cortex [13], [14] and is in contrast with models that invoke independent factors to regulate the size of the cortical representation [15], [16].

Additional insight into this issue comes from studies on albinism. The albino retina generally has an underdeveloped fovea and the cortical volume of the foveal representation is greatly reduced in these individuals [17]. It has been suggested that the cortical area is smaller because foveal underdevelopment leads to ganglion cell loss; this is supported by the narrowing of the width of the optic nerves, chiasm and tracts observed in the albino retina [17]. In contrast, Adams & Horton [18] mapped the cortical representations of angioscotomas in the squirrel monkey and found that more cortical tissue is allocated for macular ganglion cells as opposed to peripheral ones, consistent with a model in which an additional magnification is applied. As such, the very fundamental issue of what drives the organization of the primate visual cortex remains unknown. Carriers of BCM have a reduced number of cones due to disruptions in expression of normal L- and M-cone photopigment, and represent a unique example of cone loss without overt retinal disease that allows us to examine how developmental disruptions at the level of the retina affect visual function and downstream organization of the visual system.

BCM is a condition where L- and M-cone function is absent [19], due to either a mutation within the L or M genes themselves or a deletion of essential cis-regulatory DNA elements needed for transcription of photopigment genes [20]–[23]. As the L- and M- cones comprise about 95% of the entire cone mosaic, affected males have very poor acuity, myopia, nystagmus, and minimally detectable cone ERG responses. Due to the X-linked nature of the condition, female carriers are spared from a full manifestation of the associated defects and are usually indistinguishable from normal observers on most tests of visual function. However, upon careful examination, some BCM carriers have been shown to have abnormal color vision, delayed dark adaptation [24], [25], and macular changes outside the normal spectrum [20]. Some BCM carriers also have abnormal cone ERG amplitudes [25], [26] and abnormal fixational eye movements [27].

We previously examined the topography of the cone mosaics of female carriers of BCM using adaptive optics (AO) retinal imaging methods and demonstrated that cone density was reduced and mosaic regularity was disrupted in these individuals, although to a variable degree [21]. These cone mosaic abnormalities are due to degeneration of the L- and M- cones, as a result of either missing or mutated L and M photopigment. As this degeneration is thought to occur early in development [21], downstream organization of the visual system might also be expected to be disrupted as a result. It has been shown previously that significant cortical reorganization can occur in other inherited photoreceptor abnormalities [28]. To examine how cone loss disrupted the organization and function of the visual system in carriers of BCM, we tested visual function in several BCM carriers using a variety of techniques and examined the cortical organization of BCM carriers using fMRI-based retinotopic mapping. Visual function testing across the fovea in AOSLO, coupled with measurements of cone spacing from simultaneous retinal imaging allowed us to make predictions about retinal circuitry in BCM carriers and estimate retinal ganglion cell density. We combine this with fMRI-based measurements of the area of cortical visual regions, and in particular that of the foveal confluence, to question whether the downstream organization of the visual system is modulated by cone density.

## Materials and Methods

### Participants

Participants provided written informed consent after the nature and possible consequences of the study were explained. All research adhered to the tenets of the Declaration of Helsinki. Study protocols were approved by institutional research boards at the University of Rochester, Medical College of Wisconsin, and University of California, Berkeley.

#### BCM carriers

BCM carrier participants were females recruited from families with males affected with BCM. Six female BCM carriers participated in these experiments, four of whom: JC_1041, JC_1043, JC_1044, and JC_1045, correspond to participants A-III-8, B-V-2, B-V-6, and B-IV-7 in a previous study [21]. All BCM carriers except JC_1045 participated in Experiment I, three BCM carriers participated in Experiment II (JC_1041, JC_1043 & JC_1045), and four BCM carriers participated in Experiment III (JC_1041, JC_1044, JC_0120, & JC_0121).

DNA analysis was used to determine carrier status and is described in detail elsewhere [21], [29]. Four of the carriers (JC_1041, JC_1043, JC_1044, JC_1045) have a large deletion in the LCR region of one of their L/M opsin gene arrays, resulting in an absence of L and M opsin expression in cones in which that X-chromosome is active [21]. The other two (JC_0120 & JC_0121) had an L/M opsin gene array in which there was a single gene encoding a mutant opsin, shown previously to result in cone degeneration [29]. Despite these genetic differences, all six individuals are expected to have early degeneration of a subset of their L/M cones; previous imaging results with AO have shown similar cone mosaic disruptions in all BCM carriers [21], [30].

#### Controls

Many normally sighted individuals served as controls for the various experiments. For experiment I, visual resolution was measured in 16 individuals (mean age: 25.3 years; 5 males, 11 females), including one of the authors (RA). Monocular measurements from 23 eyes are used here for comparison (10 subjects had just their right eye tested, six others had both eyes tested and one had their right eye tested twice). Ten individuals (mean age: 38.7 years; 5 males, 5 females), including 3 of the authors (RA, DRW & JC) served as controls for the contrast sensitivity measurements (9 had both eyes tested; one had their left eye tested twice (DRW)). One of the authors (JC) served as a control for experiment II; we also compare our results from experiment II with results from a previous study by two of the authors (EAR & AR) that examined 5 normal subjects using nearly identical methods [31]. Ten individuals (mean age: 21.1; 8 males, 2 females) served as controls for Experiment III; we also compare our results from experiment III to the retinotopic mapping study of Dougherty and colleagues [6].

### Experiment I: Visual Function without Adaptive Optics

#### 1.1.1 Visual resolution measurement with spectacle correction only

High contrast letter resolution acuity was measured at the preferred retinal locus of fixation (PRLF) and at two eccentric locations in the temporal retina (2.5° & 8°) using a four alternative forced-choice (4AFC) tumbling E test. In a dark room, sitting at a chin rest, subjects monocularly viewed dark letters on a white background (Weber contrast ≈ –1) at a viewing distance of 310 cm through their best spectacle correction (sphere and cylinder correction only). Threshold estimation was performed by QUEST [32] with threshold set at the 82.5% correct level. QUEST was implemented in MATLAB using the Psychophysics Toolbox extensions [33], [34]. Each threshold measurement was obtained with a run of 50 trials; reported thresholds are the mean of two QUEST runs. Subjects initiated each trial with a keyboard press and after a delay of ∼200 msec the stimulus (a Snellen E) was presented in one of four orientations (up, down, left, or right). Stimulus presentation duration was 500 msec after which the subject made their response using the keyboard. For eccentric test locations, observers viewed a small circular fixation target while the Snellen Es where presented eccentrically. An ASL model 504 eye tracker (Applied Science Laboratories, Bedford, MA) was used to monitor eye position for all non-foveal trials to check for cheating eye movements. Less than 1% of trials were discarded due to subjects making eye movements away from the fixation point toward the Snellen E.

#### 1.1.2 Contrast sensitivity measurement

Contrast sensitivity was measured using a two interval forced choice (2IFC) paradigm. The stimulus was displayed on a Mitsubishi Diamond Pro SB CRT (NEC-Mitsubishi Electronics Display of America, Inc., Itasca, IL) with 1280×1024 resolution and 100 Hz refresh rate. In a dark room, subjects were seated at a chin rest a distance of 150 cm from the CRT. The stimulus, a Gabor patch, was displayed in one of two intervals (60 msec presentation duration; 500 msec ISI); background mean luminance was 17 cd/m^2^. Seven spatial frequencies (1.5, 3, 6, 9, 12, 15 & 18 cycles per degree (cpd)) were tested in separate blocks presented in randomized order. The size of the Gabor patch co-varied with spatial frequency. Contrast threshold was determined using methods described in detail elsewhere [35]. Briefly, contrast was modulated in 0.1 log unit steps in a 3-up, 1-down staircase procedure to converge at a 79% correct threshold. The number of trials to obtain each measurement varied by subject and condition, but was typically around 50. Feedback was given for incorrect responses.

### Experiment II: Visual Resolution after Adaptive Optics Correction of Ocular Aberrations

#### 2.1.1 Dual-beam retinal imaging & stimulus delivery in AOSLO

The AOSLO used in this experiment was the Berkeley second generation microelectromechanical systems (MEMS) deformable mirror (DM) based instrument, described in detail elsewhere [36], [37]; only relevant system details and specific settings pertaining to this experiment will be discussed herein. This AOSLO allows for simultaneous imaging and retinal stimulation with multiple wavelengths of light [36]–[38]; this multi-wavelength capability was utilized to project a high contrast AO-corrected tumbling E stimulus onto the retina with visible light, while simultaneously imaging the retina with infrared (IR) light. The stimulus was scanned onto the retina in a raster fashion with a 680 nm diode laser while the surrounding retina was imaged with a super luminescent diode (Superlum BroadLighter, S840-B-I-20) with mean wavelength of 840 nm and spectral FWHM of 50 nm. Vertical scan amplitude was set using digital computer control; horizontal amplitude was set manually. Field size was 48 arcmin (H)×54 arcmin (V). The central 20×20 arcmin area was optimized to be within the linear portion of the sinusoidal raster scan. The central 20×20 arcmin section consisted of ∼8 stimulus lines (pixels) per arcmin with linearity to within ∼1 pixel. An acousto-optic modulator (AOM) (Brimrose Corp., Baltimore, MD) controlled beam intensity independently for each light source. A dual-beam light delivery mode was employed whereby both lasers were modulated to be on during the forward scan and off during the return scan. At those pixel locations within the field where the stimulus was present, the AOM switched both beams off simultaneously (the stimulus thus appeared in negative contrast as black on a bright red background).

The retinal illuminance of the 680 nm stimulating light was ∼4.5 log Trolands (laser power of 0.7 µW over an area of 0.72 deg^2^). The retinal illuminance of the background imaging light was ∼2.4 log Trolands (laser power of 160 µW over an area of 0.72 deg^2^) [39]. The stimulating light, although appearing bright to the observer, was far too dim to form an image at the detector, so simultaneous modulation of the imaging beam was used to place a fiducial mark into the IR imagery. This allowed for the exact cones stimulated during a given trial to be identified (after compensating for a translational shift in the focus of each wavelength due to transverse chromatic aberration (TCA), see below). Because the visual system is much more sensitive to the stimulating wavelength (680 nm) and adapted quickly to the level of retinal illuminance, the observer could not detect the background IR light or the stimulus fiducial mark in the IR imagery.

Since two different wavelengths were used in this experiment, the effects of the chromatic aberration of the eye must be considered. TCA caused the visible stimulus and the fiducial mark to be slightly offset laterally, such that the retinal location of the stimulus in the visible wavelength was filled with IR light (see chromatic aberration compensation and measurement, below). This did not interfere with the extremely high contrast of the AO-corrected stimulus; the ratio of retinal illuminance between wavelengths yielded a Weber contrast of –0.99. Observers adapted rapidly to the bright field and had no problem performing the task comfortably. Although we previously showed that normal observers achieve similar resolution levels with either wavelength [31], 680 nm stimulating light was chosen for this study because of the multi-wavelength capability of this system, and to eliminate any possibility of the stimulus being too dim for the BCM carriers to achieve their best performance on the psychophysical test.

#### 2.1.2 Adaptive optics correction of ocular aberrations

Spherical and cylindrical lenses placed into the AOSLO system near the spectacle plane (∼14 mm from entrance pupil) corrected low order aberrations. The AOSLO measured monochromatic aberrations over a 6 mm pupil with a Shack-Hartmann wavefront sensor (SHWS) and used a 3.5 µm stroke, 140 channel MEMS-DM (Boston Micromachines Corp., Cambridge, MA) for aberration correction. AO corrected ocular aberrations at the beginning of each threshold measurement and again whenever the experimenter (monitoring image quality and RMS wavefront error) or observer (viewing the stimulus) noticed that the image or stimulus quality had degraded. To ensure that the stimulus was focused on the outer segments of the cone photoreceptors, the MEMS-DM was used to subjectively refract each subject after AO correction but prior to threshold measurement, as explained elsewhere [40]. Most subjects required no fixed defocus compensation, and when they did it was very small (∼0.05 Diopters). Locations were consecutively (not randomly) imaged, because a chromatic aberration calibration video needed to be obtained at each test location for each observer (see chromatic aberration compensation and measurement, below). An AO correction was typically stable for around 10–30 trials, depending upon the observer.

#### 2.1.3 Resolution threshold estimation

High-contrast photopic letter acuity was measured using a 4AFC tumbling E test at the PRLF and at several locations along the horizontal temporal meridian within the central fovea (0–2.5°). For eccentric test locations, subjects viewed a fixation target off a pellicle beam splitter placed into the AOSLO between the spectacle lenses and the first system mirror. Both eyes of one BCM carrier (JC_1045) were tested, while one eye was examined for all other observers (typically the right eye; see table 1). During imaging and psychophysics, the fellow eye was occluded with an eye patch. Similar to resolution testing without AO in Experiment I, threshold estimation was performed by QUEST [32] with threshold set at the 82.5% correct level. QUEST was implemented in MATLAB using the Psychophysics Toolbox extensions [33], [34]. Each threshold measurement was obtained with a run of 40 trials. Each subject was given a practice run at their PRLF, and then between three and six threshold measurements were made at each of several foveal locations along the temporal horizontal meridian. Thresholds shown are the average of all measurements made at each location. Subjects initiated each trial with a keyboard press and after a short delay the stimulus was presented in one of four orientations (up, down, left, or right). Stimulus presentation duration was 500 msec, after which the subject made their response using the arrow keys of a computer keyboard. It should be noted that this stimulus duration is shorter than the 1000 ms trials used in a similar experiment on normal observers [31], but identical to that used to assess visual resolution after AO correction in emmetropes and low myopes [40]. This duration was chosen because it reduced the amount of light exposure to the retina, decreased total imaging session time (as limited time was available for imaging and psychophysics on these observers), reduced observer fatigue, and because it corresponded to the duration used for resolution tests without AO-correction in Experiment I. Pilot testing with normal observers at eccentric test locations showed no difference in measured thresholds with either a 500 ms or 1000 msec duration. Westheimer tested the effect of stimulus duration on visual acuity and determined that resolution acuity improved with durations up to 400 ms and perhaps longer [41]; the stimulus duration used herein (500 ms) therefore exceeds the critical duration of all areas tested [42].

**Table 1:** Transverse chromatic aberration.

Subject	Eye	Distance from PRLF (degrees)	Horizontal (arcmin)	Vertical (arcmin)
JC_1041	(OD)	0	2.752	0.500
		0.864	2.585	0.334
		1.92	2.585	0.417
		2.50	2.585	0.417
JC_1043	(OD)	0	3.082	1.627
		0.861	2.911	1.798
		2.649	3.767	0.685
JC_1045	(OS)	0	2.576	0.499
		2.047	2.244	0.748
	(OD)	0	2.305	0.854
		0.941	2.305	0.854
Control	(OD)	0	2.704	0.082
		1.103	2.377	0.025
		2.439	3.852	0.029

Measured difference in lateral focus (TCA) between imaging and stimulating wavelengths.

#### 2.1.4 Retinal imaging of areas surrounding resolution test locations and processing of retinal imagery

To build a continuous map of the photoreceptor mosaic across test areas, several videos acquired prior to and during psychophysical testing were combined. For images taken prior to resolution tests, the field size and other imaging parameters were the same as those listed above; retinas were imaged solely in 840 nm light for videos acquired prior to psychophysical testing. The PRLF was imaged first and then the fixation target was repositioned such that overlapping retinal areas could be imaged, extending temporally from the PRLF out to between 2 and 3 degrees, depending upon the observer. The temporal retina was chosen to facilitate comparison to a previous study of normal observers [31].

Digital AOSLO videos of test locations and neighboring retinal areas were processed using methods described previously [21], [31]. Cone positions were localized using a combination of automated [43] and manual methods [21], [31]. Inter-cone distance (ICD), the average center-to-center distance between a cone and each of its nearest neighbors, and the Nyquist limit of the cone mosaic (N_c_) were measured from localized cones using previously described methods [31]. Stimulated retinal areas were determined from the retinal imagery using cross-correlation methods implemented in MATLAB and described in detail elsewhere [31]. For comparing N_c_ and AO-corrected visual resolution (MAR_AO_) at resolution test locations, N_c_ was averaged over an elliptical area subtending ±2 SD of the position of the stimulus on the retina during the resolution measurement at each test location [31].

#### 2.1.5 Chromatic aberration compensation and measurement

For multiple wavelength imaging in AOSLO, the chromatic aberration of the eye causes different wavelengths of light to focus at different lateral and axial locations on the retina [38], [44], [45]. To deliver an optimally focused stimulus after AO correction (when stimulating with 680 nm light and imaging the retina with 840 nm light) longitudinal chromatic aberration (LCA), the difference in focus between wavelengths, must be compensated for. This was accomplished through manual adjustment of the 680 nm source in a calibration phase prior to the combined imaging/psychophysics session; published estimates of the LCA of the eye between these two wavelengths were used as a starting point [38]. The AOSLO has a dedicated PMT detector for each wavelength, allowing video imagery obtained in each wavelength to be acquired simultaneously. To accomplish LCA compensation, the AO loop was first closed on the 840 nm imaging wavelength alone, bringing that image into sharp focus. The 680 nm source was then activated (at a power level sufficient to form an image at its detector; ∼30 µW; retinal illuminance of ∼6.2 log Trolands; <1% ANSI maximum permissible exposure) [46]. Both video streams were simultaneously presented to the experimenter on a CRT computer monitor in real time. Using image quality as a subjective metric, the 680 nm source was adjusted to come into sharp focus at the same focal plane as the 840 nm image.

**Figure 1:**
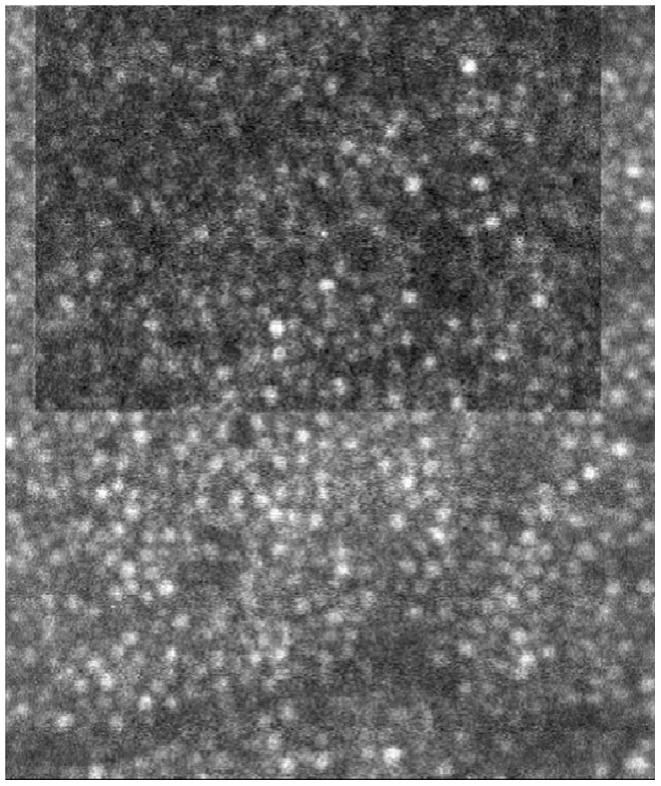
TCA measurement. Single frame of a calibration video used to measure transverse chromatic aberration (TCA). Upper rectangular region (appearing darker) was imaged using 680 nm light while the surrounding area was imaged with 840 nm light. The position of the upper region translated with temporal wavelength alternation, encoding the TCA and allowing the shift to be measured algorithmically.

The other type of chromatic aberration, transverse chromatic aberration (TCA), causes a lateral shift of the retinal images formed by each wavelength [47]. TCA was measured to precisely determine the lateral shift between wavelengths and thus which cones were stimulated. This was accomplished by acquiring a special calibration video of the retina for use in offline TCA calculation. This calibration video was created by using the AOM to alternate at 2 Hz between a full field of 840 nm light and a field which contained a window of 680 nm light within the 840 nm light field. This resulted in a video in which a patch was alternately imaged with each wavelength, while the surrounding area was continuously imaged with 840 nm light. The area alternately imaged with both wavelengths appeared to shift when the wavelength switched (due to TCA) while the region imaged with the single wavelength appeared normally (a single frame from one of these calibration videos is shown in Figure 1). TCA was calculated from the calibration videos using custom MATLAB software written by one of the authors (EAR) that read in the digital video file and prompted the user to manually select first the IR only region, and then the alternating red/IR region. The user then selected the first red frame, which set the phase of the wavelength alternation. The video was then stabilized at the frame rate with respect to the IR only region. Stabilization was performed by taking the peak of the FFT cross-correlation function. This resulted in a new video that was stabilized at the frame rate with respect to the IR imagery. The stabilized video was then reprocessed using the same methodology, but the stabilization area was chosen to be the area that alternated between red and IR. The analysis software computed a motion trace for each; the difference between these two motion traces encoded the TCA shift. Vertical and horizontal shifts were each averaged with respect to the phase of the wavelength alternation. Due to unavoidable errors in the stabilization process that arose due to large eye movements or blinks, some of the raw TCA traces contained errors. These errors were compensated for by averaging several cycles of the TCA shift and removing those spurious shifts which fell beyond two standard deviations of the mean. The result was two square wave traces (one horizontal and one vertical) which encoded the TCA and thus the translation required to bring the red and IR regions into register. The compensatory translational shifts were then applied to the imagery and displayed to the observer with the two (now overlapping) images alternately presented such that the experimenter could visually inspect the calculated TCA shifts to confirm that the calculated shifts appropriately placed the two images in register. Measured TCA for each subject is listed in Table 1. The measured TCA was a combination of ocular and system TCA, but is likely dominated (especially in the horizontal direction) by a lateral misalignment between the red and IR light sources. Although it is expected that TCA will change to some extent with changes in pupil position, it is expected that for a given psychophysics test location the TCA change due to small shifts of pupil position will be insignificant with respect to our results. It should be noted that chromatic aberration also causes a chromatic difference in magnification (CDM). The effect of CDM is very small (<1% between 400 and 700 nm) and considered to be negligible with respect to these results [38], [48]–[50].

#### 2.1.6 The size of retinal features in AOSLO images

Magnification induced by trial lenses used for some subjects in the AOSLO was calculated and applied to all AO measurements, as explained elsewhere [40]. Bennett’s adjusted axial length method was used to calculate the size of retinal features [51]. The only biometric measurement required for this method is the axial length of the eye, which was measured optically with an IOLMaster (Carl Zeiss Meditec, Inc., Germany). Further details on calculations and inherent error estimates can be found elsewhere [40]. Axial lengths, spherical equivalent of spectacle lenses used during retinal imaging, resulting spectacle magnifications, microns per degree of visual angle and microns per pixel of retinal imagery are listed in Table 2.

**Table 2:** Biometry and AOSLO imaging parameters.

Subject	Eye	Axial Length (mm)	Spherical Equivalent of Spectacle Lenses (D)	Spectacle Magnification (%)	*m* (µm/degree)	AOSLO Image Resolution(µm/pixel)
JC_1041	(OD)	23.84	−3.375	95.5	274.61	0.4186
JC_1043	(OD)	22.51	−0.25	99.7	269.30	0.3870
JC_1045	(OS)	22.66	−0.5	99.3	270.28	0.3798
	(OD)	22.57	−0.5	99.3	269.11	0.3888
Control	(OD)	27.46	−4.25	94.4	316.03	0.4841

Axial length, spherical equivalent of spectacle lenses, magnification, distance relations, and image resolution.

### Experiment III: Retinotopic Mapping of the Visual Cortex

#### 3.1.1 Data acquisition

A 3T Siemens Trio MRI system (Siemens Medical Solutions, Erlangen, Germany) housed at the Rochester Center for Brain Imaging was used for all cortical imaging. For functional imaging we used gradient echo, echo planar imaging (EPI): 30 ms TE, 90° flip angle, 3 mm slice thickness, 24 cm FOV, 32 slices, 64×64 matrix for a 3 mm in-plane resolution, 3 sec TR for a total of 84 time points. A set of 2D fast spin-echo images was acquired before the series of functional scans. These T1-weighted slices were placed in the same physical orientation as the functional scans and used to align the functional data with the high-resolution structural dataset [52]. Two sets of high-resolution anatomical images were obtained using a 3-D MPRAGE pulse sequence with 1 mm^3^ isotropic voxels. As a result of their enhanced grey/white matter contrast, these images allowed for precise localization of functional activation.

#### 3.1.2 Stimuli and procedure

Standard retinotopic mapping techniques [53]–[56] were used to create polar angle and eccentricity maps of the visual cortex. We used a rotating 90° wedge and expanding annuli sections of a black and white radial checkerboard to create both polar angle and eccentricity maps of the visual cortex. The visual stimulus had a fixation target spanning from 0–0.25° to ensure that the subjects maintained their gaze at the center. The radial checkerboard was set at 100% contrast, was contrast-reversing at 4 Hz, and subtended a visual angle that extended from 0.25 to 12 degrees. Each stimulus rotated or expanded at a rate of 6 cycles per scanning run (one cycle every 45 s). Each subject completed 4 runs each of the expansion and rotation stimuli. Some scans were repeated if the subject informed the experimenter that they had moved during the run. Head motion was minimized by keeping the runs short (about 4 min), packing the head and neck with foam pads, and repeatedly instructing subjects not to move. To maintain fixation and attention, subjects were given a task monitoring changes to the central fixation spot. The stimuli were back-projected to subjects via an LCD projector fitted with a special focusing lens. Subjects viewed the display by way of a mirror positioned at ∼45° above the eyes and pointed towards the translucent screen positioned on the back of the scanner.

#### 3.1.3 Retinotopic mapping analysis

The first 4 time frames of each functional run were discarded due to start-up magnetization transients. The remaining reconstructed functional images were corrected for slice acquisition timing and motion artifacts across all runs using the Stanford mrVista toolbox. We followed the analysis steps used by Dougherty et al. [6] with the exception that the borders between the different visual areas were drawn by hand instead of using an automated algorithm. Manual boundary delineation for all subjects was performed by one of the authors (AG). We were not able to implement an automated delineation method because in contrast to the Dougherty study, where highly experienced subjects were scanned many times and data were averaged to obtain very high signal-to-noise ratio images amenable to automated analysis, the data here were obtained in a single imaging session with relatively inexperienced participants. Subsequently, our maps contained rather jagged phase-reversal boundaries rather than straight lines. Despite the inherit bias that may exist from manual delineation of the boundaries between neighboring visual areas we are confident that our measurements describe the data as accurately as possible.

#### 3.1.4 Identification of early visual areas

The expanding annuli stimulus allowed us to delineate central from more peripheral regions; the rotating wedge allowed us to separate the boundaries between V1, V2 and V3, as reversals are observed in the boundaries between these areas. We defined the region of foveal confluence as the intersection between a mask of the central 2° of eccentricity and the region including the central representations of early visual areas. Using brain segmentation and cortical flattening algorithms, we delineated the location of retinotopic areas for each subject. We used the Stanford-developed software mrGray and mrVista tools to semi-automatically classify grey and white matter within the occipital lobe and then to unfold it, with minimal distortion, into a flat sheet [55], [57]. Typically, an area of 6 cm radius is unfolded from around a point within the fundus of the calcarine sulcus and 3 cm anterior to the occipital pole. The software returns coordinate arrays that map points in the flattened representation to the anatomical volume. For each retinotopic dataset, magnitude and phase images of the fundamental Fourier component are created. These images are transformed and re-sampled to the same space as the classified grey/white matter volume. Using the point-to-point correspondence derived from the flattening, each point in the phase image with a magnitude above a certain threshold is plotted onto the flat map. Horizontal and vertical meridia were identified from the maxima and minima in plots of the absolute cosine of the mapped polar angle. All visual areas were then restricted to phases corresponding to eccentricities greater than 2° and less than 12°. All surface area measurements were made on the 3D cortical manifold based on ROI’s predefined on the 2D flat map [58]. The different visual areas and resulting foveal confluence were then delineated by hand [6].

## Results

### Experiment I

**Figure 2:**
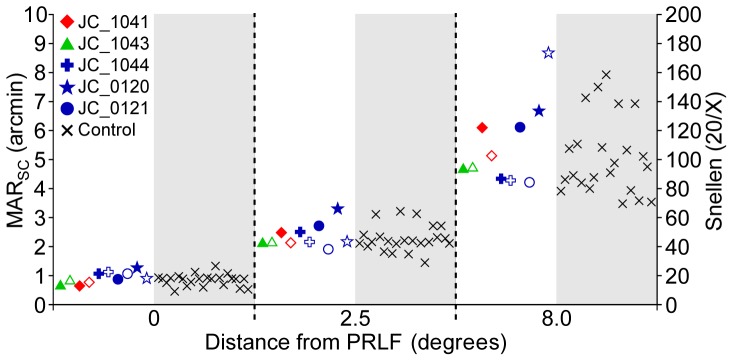
Spectacle corrected visual resolution (MAR_SC_) as a function of eccentricity. Resolution thresholds for 10 BCM carrier eyes are compared to 23 normal eyes at the preferred retinal locus of fixation (0°), 2.5° and 8°.

**Figure 3:**
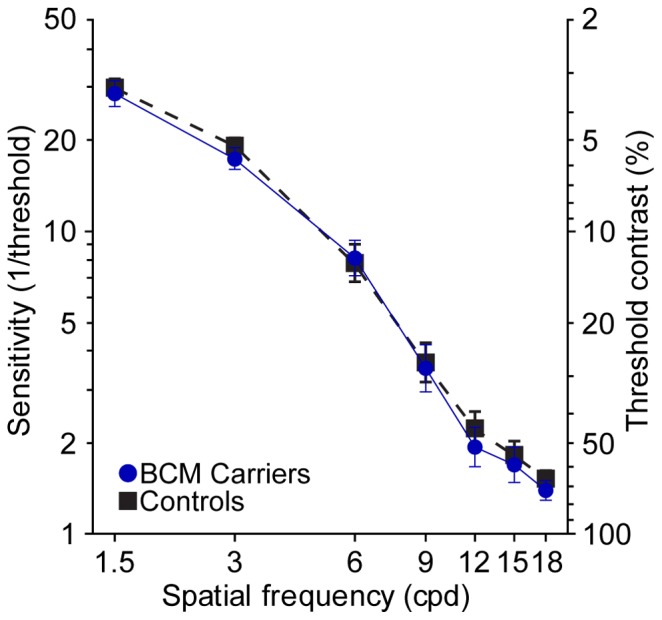
Contrast sensitivity functions. The mean of 5 BCM carriers (blue circles; solid lines) are compared to 10 age matched control observers (black squares; dashed lines). Data points represent the mean of the threshold contrast at each spatial frequency for all eyes tested in each group (BCM carriers, n = 10; Controls, n = 20). Error bars are ± SEM of threshold contrast.

Visual acuity measurements with spectacle correction only (MAR_SC_) for 10 BCM carrier eyes (both eyes of JC_1020, JC_1021, JC_0141, JC_0143 & JC_0144) are compared to measurements obtained from controls in Figure 2. The mean MAR_SC_ was 0.92 acrmin (SD: 0.21; n = 10) at the PRLF for the BCM carriers and 0.82 arcmin (SD: 0.2; n = 23) for the control eyes. Mean MAR_SC_ was 2.37 acrmin (SD: 0.4; n = 10) for the BCM carriers and 2.26 arcmin (SD: 0.45; n = 23) for the control eyes at 2.5 degrees. At 8 degrees the mean MAR_SC_ was 5.51 acrmin (SD: 1.42; n = 10) for the BCM carriers and 5.08 arcmin (SD: 1.33; n = 23) for the control eyes. MAR_SC_ for all subjects was similar to that expected at the eccentricities tested for normally sighted persons [8]. Although the mean of the BCM carrier group was consistently slightly higher than that observed for the control group, no significant difference was found between groups at the PRLF (p = 0.1913; t-test, two sample; d = 0.5031), the 2.5° eccentric location (p = 0.5103; t-test, two sample; d = 0.2587), or 8° eccentric location (p = 0.4097; t-test, two sample; d = 0.3123). Contrast sensitivity functions for the same 5 BCM carriers and ten controls are shown in Figure 3. For all subjects, left and right eyes were tested monocularly at each spatial frequency; each data point in Figure 3 is the mean threshold contrast for all eyes tested in each group. No difference was found between the mean threshold contrast of the BCM carriers and the control group at any of the spatial frequencies tested; results in both groups were similar to those found previously in normal eyes [35].

### Experiment II

#### 2.2.1 Cone spacing and the Nyquist limit of the cone mosaic (N_c_)

**Figure 4:**
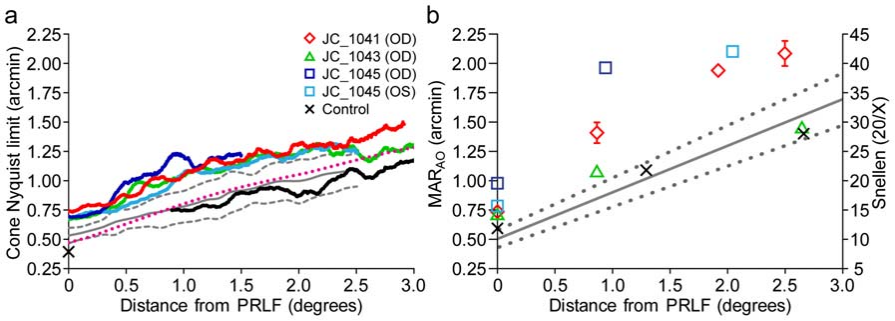
The Nyquist limit of the cone mosaic and adaptive optics corrected visual resolution. a) Nyquist limit of the cone mosaic (N_c_) as a function of eccentricity. Lines colored after symbols in inset legend. The black X is the estimate of N_c_ converted from density of control eye measured in a previous study (Putnam et al., 2005). Dotted magenta line shows mean N_c_ derived from mean of densities reported by Curcio et al. (1990b). Solid and dashed grey lines are mean and ±2 SD of 6 normal eyes (the control eye and the 5 eyes from Rossi & Roorda, 2010). b) Adaptive optics corrected visual resolution (MAR_AO_) as a function of eccentricity. Minimum angle of resolution (MAR) is shown on left ordinate, while equivalent Snellen acuity is shown on the right. Symbol-subject relations are provided in inset legend. Solid and dashed lines are regression line and 95% confidence intervals fit to the data of 5 normal observers from Rossi & Roorda (2010).

Cone spacing at the PRLF was larger than normal in BCM carrier eyes. The average minimum ICD measured from the four carrier eyes was 0.79 arcmin (SD = 0.03). JC_1041 had the largest minimum ICD of 0.84 arcmin. JC_1045 had minimum ICD of 0.79 arcmin in each eye. JC_1043 had the lowest ICD, of 0.76 arcmin. Minimum ICD was found less than 3 arcmin from the PRLF for all carrier eyes. An assessment of mosaic regularity confirmed a fairly triangular packing arrangement in the carrier retina, indicating that this was probably an appropriate method for calculating N_c_ [21]. N_c_ across the horizontal temporal retina is shown in Figure 4a. The mean N_c_ and mean ±2 SD of six normal observers (the control eye and 5 from a previous study [31]) and mean N_c_ calculated from the density measurements of Curcio [1] are shown for comparison. Densities from Curcio et al. were converted to N_c_ using the following equation:
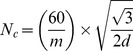
(1)Where *m* is the number of mm per degree and *d* is cone density. A value of *m* of 0.289 was used to convert the data, and assumed that all eyes were emmetropic with 24 mm axial length.

Minimum N_c_ was 0.68, 0.68, 0.66 and 0.73 arcmin for JC_1045 (OD), JC_1045 (OS), JC_1043 and JC_1041, respectively. Mean minimum N_c_ was 0.69 arcmin (SD = 0.029) for the four carrier eyes. N_c_ was estimated for the control eye based upon a cone density measurement obtained previously [59]. Since it was previously shown that the PRLF of the control eye was displaced from the position of peak cone density to an area where density had fallen to ∼10% below its peak [59], peak density was first reduced by 10% to estimate the density at the PRLF and then converted to N_c_ using the same equation used for the data of Curcio et al., but with the appropriate *m* value for this subject (in Table 2). This resulted in an estimated N_c_ of 0.39 arcmin at the PRLF for the control eye.

#### 2.2.2 AO-corrected visual resolution and N_c_

**Figure 5:**
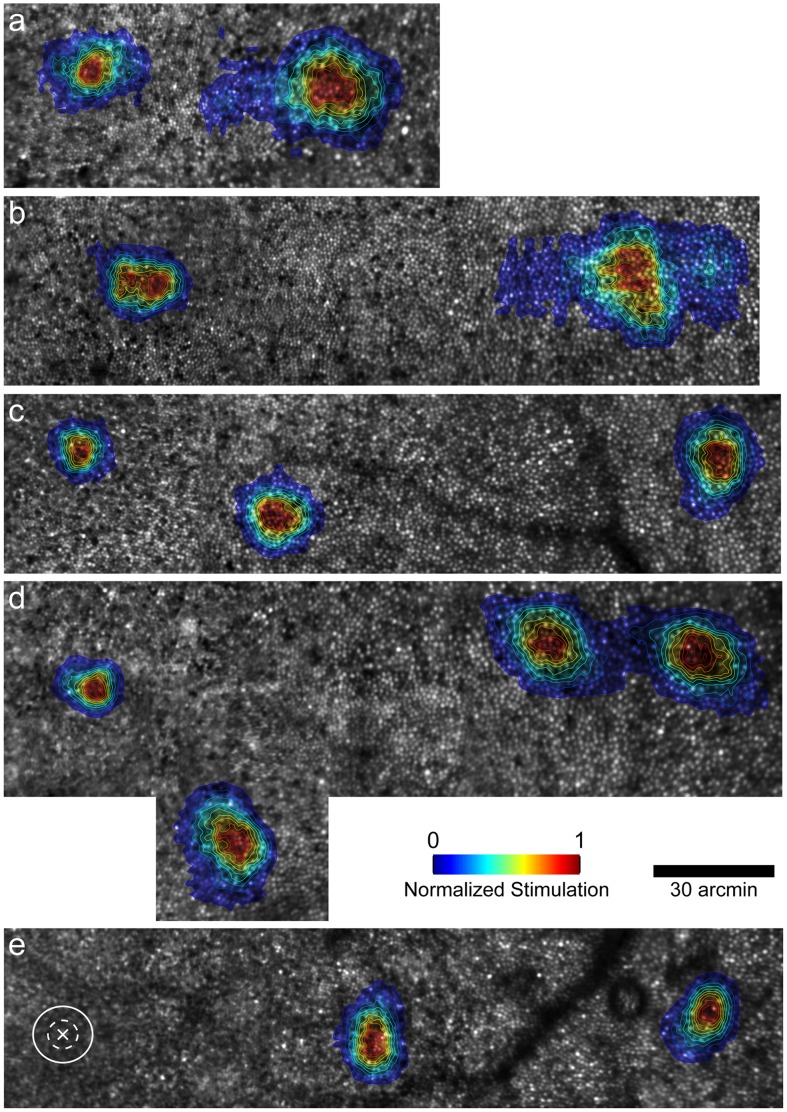
Retinal imagery overlaid with contour maps showing stimulated cones. Topographical contour maps overlaid in color show the normalized level of cone stimulation at each test location. a) JC_1045 (OD); b) JC_1045 (OS); c) JC_1043; d) JC_1041; e) control. Cones appear as bright circles. Each cone stimulated over the course of the psychophysical tests was localized on the mosaic using methods described previously (Rossi & Roorda, 2010). Color bar shows normalized level of cone stimulation; PRLF is the location at the far left of all images. Since cones were not resolved at the PRLF for the control eye, an X marks the PRLF, with the solid and dashed white ellipses drawn to show ±1 and ±2 SD of stimulated area, respectively.

AO-corrected visual resolution (MAR_AO_) is plotted versus eccentricity in Figure 4b. Cone mosaics overlaid with topographic maps of stimulated cones are shown in Figure 5. As can be seen from Figure 5, the stimuli did not always fall precisely along the horizontal meridian, so MAR_AO_ is plotted versus linear distance from the PRLF in Figure 4b. For comparison, a linear regression line fit to the data of the 5 normal observers from a previous study using similar methods [31] is plotted in Figure 4b along with 95% confidence intervals as the dashed and solid lines, respectively. The control observer fell within the normal range at all test locations; it should be noted that he was near the high end of the normal range at the PRLF. This is not surprising as this observer was myopic and it has been shown previously that myopes tend to perform worse than emmetropes in AO corrected tests of visual resolution [40].

Resolution was outside the normal range at the PRLF and at the 0.86° location for JC_1043, however her performance fell back to within the normal range by the 2.64° test location. It should be noted that she also was outside the normal range for N_c_ until ∼2.5° from the PRLF. The other carrier eyes had worse resolution than previously examined normal eyes at all test locations. JC_1045, who had both eyes tested, and had similar spacing at the PRLF in each eye had worse resolution in her right eye at the PRLF; it is interesting to note that her right eye was the one that showed the more rapid decrease in density away from the PRLF. Her performance at the 0.94° test location in her right eye (MAR_AO_ = 1.96 arcmin) was similar to that at the 2° test location in her left eye (MAR_AO_ = 2.1 arcmin), where N_c_ was similar in both eyes (N_c_ was 1.13 arcmin and 1.25 arcmin in her right and left eye, respectively).

**Figure 6:**
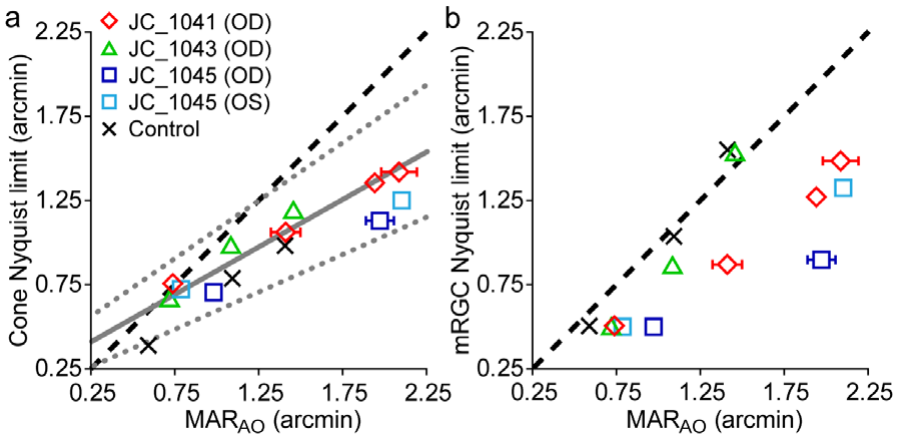
Adaptive optics corrected resolution measurements compared to cone and mRGC Nyquist limits. a) Adaptive optics corrected visual resolution (MAR_AO_) is worse than predicted by the cone Nyquist limit outside the PRLF. JC_1043, JC_1041, JC_1045 (OD), JC_1045 (OS) are shown as green triangles, red diamonds, dark blue, and light blue squares, respectively. Control is shown as black X’s. Dashed black line is the 1∶1 line of equality. Solid and dotted gray lines are linear regression line with 95% upper and lower confidence intervals from the data of 5 normal observers from Rossi & Roorda (2010). Error bars are ± SEM and omitted when smaller than the symbol. b) Nyquist limit of the normal eye’s mRGC mosaic does not predict resolution outside the PRLF for BCM carriers. Symbols are the same as in (a). Dashed black line is the 1∶1 line of equality. MAR of control eye and JC_1043 at the most eccentric test location fit well with the Drasdo model (Drasdo et al., 2007) estimates of N_mRGC_ outside the PRLF; resolution at the other test locations for JC_1043 and for the other 3 carrier eyes at all locations was worse than predicted by N_mRGC_.

MAR_AO_ is plotted against N_c_ at resolution test locations in Figure 6a. For comparison, a linear regression line with 95% confidence intervals fit to the data of 5 normal observers from a previous study using similar methods [31] are shown as the solid and dotted grey lines, respectively. MAR_AO_ agreed well with estimates of N_c_ at the PRLF for three of the four carrier eyes, consistent with results obtained from normal eyes [31]. The myopic control eye had a moderate difference between MAR_AO_ and N_c_ at the PRLF, suggesting that the resolution deficit seen in myopia may, at least in part, be attributed to postreceptoral factors [40].

**Figure 7:**
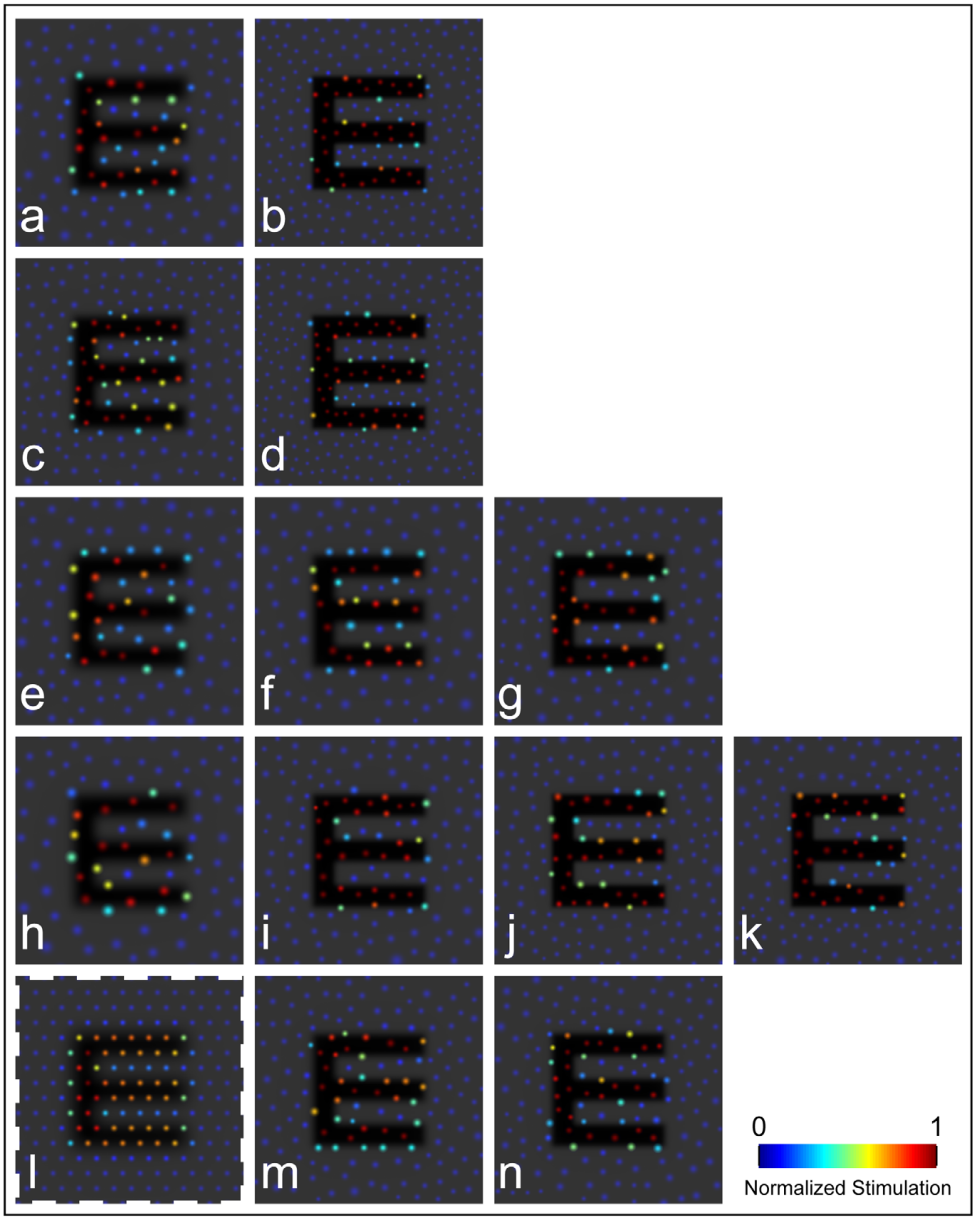
At threshold, more cones are required to see the stimulus at locations outside the PRLF. Simulated cone–stimulus interaction profiles. Each circle denotes a cone aperture; color represents the normalized level of aperture filling for a single frame of stimulus presentation. Cone locations were obtained from retinal imagery for all locations except the PRLF of the control eye, which was modeled as a triangular mosaic (l; denoted with dashed outline). Cone aperture was modeled as a 2D Gaussian, with full width at half maximum set to 34% of ICD. Cone apertures shown in blue were filled with light, while those shown in red were filled with the stimulus; color bar gives normalized level of cone stimulation (stimulus aperture filling). a) JC_1045 (OS): PRLF; b) JC_1045 (OS): 2.05°; c) JC_1045 (OD): PRLF; d) JC_1045 (OD): 0.94°; e) JC_1043: PRLF; f) JC_1043∶0.86°; g) JC_1043∶2.65°; h) JC_1041: PRLF; i) JC_1041∶0.86°; j) JC_1041∶1.92°; k) JC_1041∶2.5°; l) control: PRLF; m) control: 1.28°; n) control: 2.66°.

Outside the PRLF, MAR_AO_ decreased at a greater rate with increasing eccentricity than predicted by N_c_. This finding is in agreement with results obtained previously for normal observers [31]. Figure 7 shows simulated patterns of cone stimulation for a threshold sized stimulus located at the center of each test location for each observer. Cone apertures were estimated to be 2D Gaussians with full width at half maximum set to 34% of ICD [60] and are colored relative to the normalized level of cone stimulation. The diffraction limited PSF for a 6 mm pupil was used to blur the stimulus, which is overlaid with semi-transparent shading in Figure 7. Since cones were not resolved at the PRLF for the control eye, a simulated hexagonal mosaic with the estimated spacing for the control eye was used. For this simulation, images are normalized by the threshold letter size (each letter in the figure would be a different size if they were not) so that the number of cones sampling the E at the threshold can be compared irrespective of threshold. As can be appreciated clearly from this simulation, more cones are stimulated by a threshold sized stimulus as distance from the PRLF increases, consistent with results obtained for normal observers [31]. The control eye shown here does not precisely follow the pattern seen previously for normal eyes, as the estimated spacing from density at the PRLF suggests performance was worse than N_c_; this pattern is consistent with reduced MAR_AO_ for myopic eyes relative to emmetropic eyes seen at the PRLF previously [40].

#### 2.2.3 Relationship between MAR_AO_ and N_mRGC_ in BCM carriers suggests significant post-receptoral differences

MAR_AO_ is plotted against the estimated normal Nyquist limit of the mRGC mosaic (N_mRGC_) in Figure 6b. The model of midget ganglion cell receptive field density in the human visual field from Drasdo and colleagues [61] was used to estimate the Nyquist limit of midget ganglion cell receptive fields, using methods published previously [31]. The data points from normal observers examined in a previous study cluster around the dashed 1∶1 line of equality [31]; this expected relationship was found for the control eye. Linear regression lines fit to the data from normal observers have an average slope close to one, indicating that MAR_AO_ matches the Nyquist limit of the mRGC mosaic [31]. A linear regression line fit to the data from the control observer had a slope of 1.26, slightly steeper than the average of 1.11 of five normal eyes [31]; the increased slope for the control eye relative to the other normal observers can again most likely be attributed to the myopic shift towards reduced resolution at the PRLF, and additionally, by the small number of data points obtained.

The findings for the BCM carriers are quite different from what has been shown previously for normal observers. For JC_1043, the data points fall below the equality line at the PRLF and 0.86° test locations, with the data point for the most eccentric test location (2.65°) falling near the equality line (where she also appeared normal on all other measures). Data points for the other 3 carrier eyes fell below the line of equality at all locations, showing that MAR_AO_ is worse than predicted by the Drasdo et al. model of mRGC receptive field density from normal eyes [61]. This finding suggests significant postreceptoral differences in the BCM carriers.

#### 2.2.4 Fixation stability

**Figure 8:**
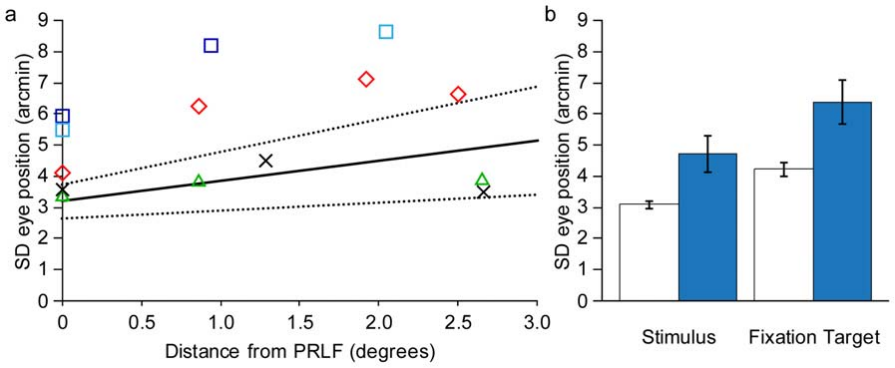
Fixation stability. a) BCM carriers with substantial cone loss had unstable fixation when compared to normal controls. SD of stimulus position averaged for vertical and horizontal directions as a function of eccentricity. Solid and dashed lines are linear regression line and upper and lower 95% confidence intervals fit to data from 6 normal eyes (control eye and 5 normal eyes from Rossi & Roorda, 2010). JC_1043, JC_1041, JC_1045 (OD), JC_1045 (OS) are shown as green triangles, red diamonds, dark blue, and light blue squares, respectively. Control eye is shown as a black X. JC_1041, JC_1045 fell outside the normal range at all locations, while JC_1043 (the carrier with the least amount of cone loss) and the control eye both fell within the normal range at all test locations. b) BCM carriers and normal controls where more stable when looking at the stimulus vs. the fixation target. Normal observers (white bars) are more stable than carriers (blue bars) in either condition. Mean of 6 normal eyes is shown (the control eye, and the 5 normal eyes from Rossi & Roorda, 2010). Mean of the 4 carrier eyes in (a) are shown. Error bars are ± SEM.

Fixation stability was assessed by precisely localizing the position of the stimulus on the retina for each frame of the AOSLO video from each psychophysical trial. This positional information precisely localized test locations for comparing MAR_AO_ and N_c_ and had the further advantage of providing a trace of eye position for assessing fixation stability. The standard deviation of the position of the stimulus on the retina at each psychophysics test location is a precise measurement of the fixational spread of the eye. The standard deviation of the stimulus position (averaged for both x and y directions) is plotted versus eccentricity in Figure 8a. For comparison, a linear regression fit, with upper and lower 95% confidence intervals is plotted for 6 normal observers (the 5 eyes from [31] and the control eye from this study). Two of the BCM carriers (JC_1041 and JC_1045) had fixational eye movements that were outside the normal range at all locations, while one (JC_1043) was within the normal limits at all test locations. Figure 8b compares the SD of eye position when observers either looked at the fixation target or at the stimulus. Fixation was more stable for both groups when looking at the stimulus than when looking at the fixation target, with the carriers showing less stable fixation than normal observers in both conditions.

### Experiment III

#### 3.2.1 fMRI-based retinotopic organization in BCM carriers

Table 3 contains the surface area measurements of the foveal confluence from the BCM carriers. Values from our normal controls and from Dougherty et al. [6] are compared to those obtained from the BCM carrier group in table 4. Measurements for the fovea were quite similar between BCM carriers (n = 4; mean = 1438 mm^2^; SEM = 143 mm^2^) and normal controls (n = 10; mean = 1495 mm^2^; SEM = 159) but were lower in both cases than those reported by Dougherty et al. [6], where the mean was 2095 mm^2^ (n = 7; SEM = 177). It should be noted that gender disparities exist between our control group and the BCM carriers – the BCM carriers are female (n = 4), whereas our controls were mostly male (n = 10); the gender of the participants in the Dougherty et al. study (n = 7), which differed the most from our measurements, were not reported [6].

**Table 3:** Surface area measurements of the foveal confluence.

Subject	Hemisphere	Area of the foveal confluence (mm^2^)
JC_1041	Left	1397
	Right	1869
JC_1044	Left	2024
	Right	1417
JC_0120	Left	844
	Right	947
JC_0121	Left	1453
	Right	1556

The area of the foveal confluence of V1, V2, & V3 for right and left hemispheres of the 4 BCM carriers.

**Table 4:** The area of the foveal confluence is similar in both normal subjects and BCM carriers.

	BCM carriers (n = 4)	Controls (n = 10)	Dougherty et al., 2003(n = 7)
Mean	1438	1495	2095
Median	1435	1603	2039
SD	403	390	638
SEM	143	159	177
Min	844	1085	982
Max	2024	2467	2940

The area (in mm^2^) of the foveal confluence of V1, V2, & V3 of 10 control eyes are compared to the results from the BCM carriers. The results of Dougherty et al. (2003) for 7 normal eyes are shown for comparison.

**Figure 9:**
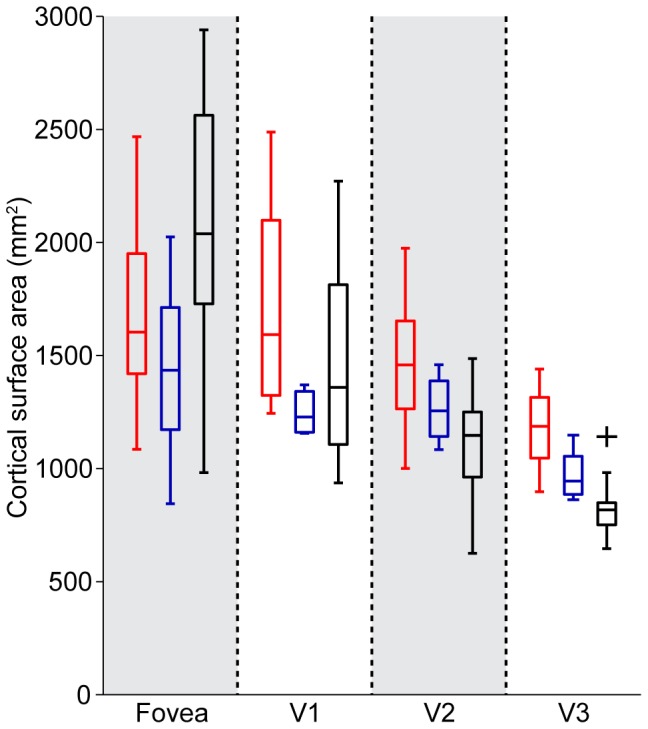
Cortical surface area in BCM carriers was similar to control data and published measurements obtained using similar methods. Surface area measurements for the foveal confluence and areas V1, V2, & V3. BCM carriers (in blue) are compared to 10 controls (in red) and to the data of Dougherty et al. (2003) (in black). For each box, the central mark corresponds to the median, the edges of the box are the 25^th^ and 75^th^ percentiles, the whiskers extend to the most extreme data points not considered outliers, and any potential outlier is plotted individually as a black cross, such as in the V3 subpanel.

**Figure 10:**
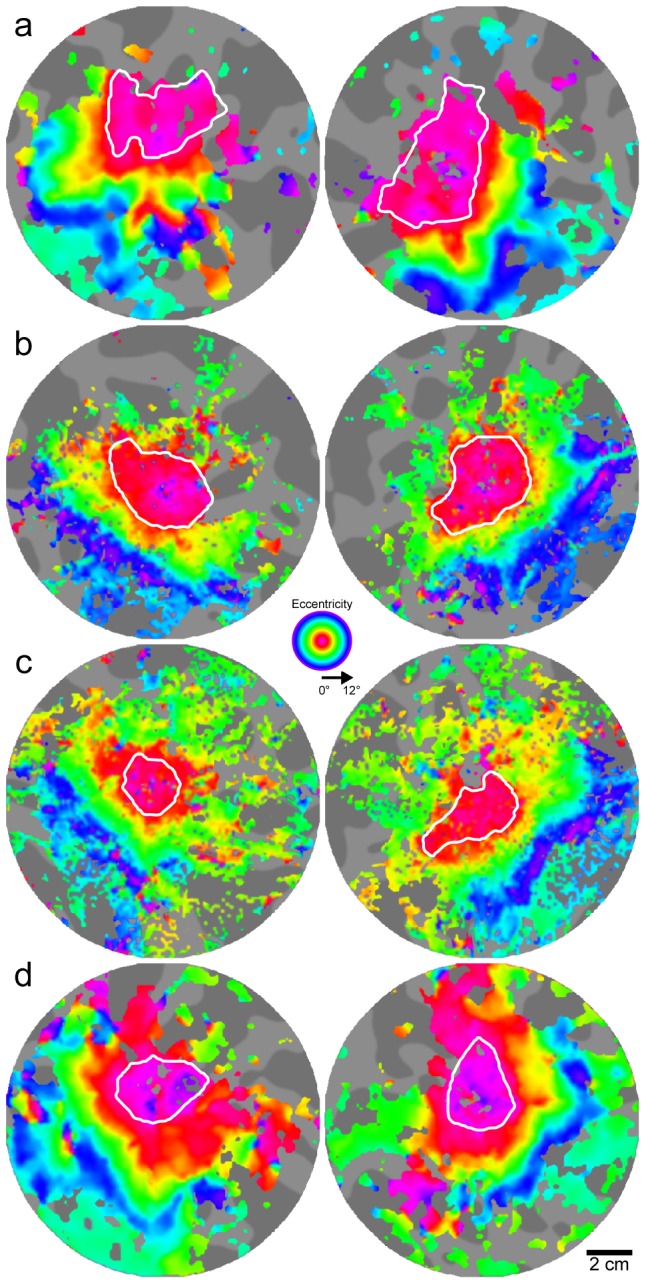
The area of the foveal confluence is similar in both BCM carriers and normal controls. Flattened pseudo-color phase maps are used to visualize retinotopic maps for: a) JC_1041, b) JC_1044, c) JC_0120, and d) control. Expanding ring stimuli were used to map retinotopic eccentricity. White contours outline the foveal confluence (0°–2°) of early visual areas. Image pairs for each subject are left and right hemispheres. Gray background shows flat map of cortical anatomy. Dark regions are sulci; bright regions are gyri. Note that because the underlying anatomy is different for each subject and hemisphere, areas are not directly comparable between images; measurements are listed in Table 2.

**Figure 11:**
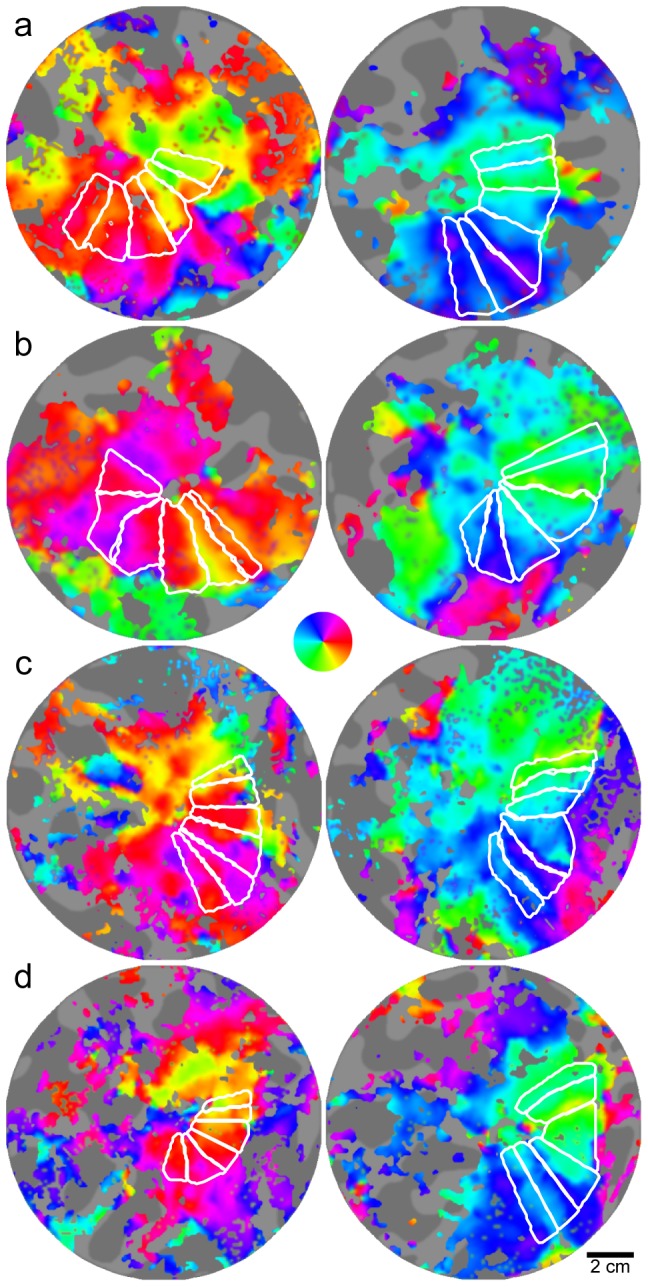
Phase maps and corresponding visual areas. Flattened pseudo-color phase maps are shown for a) JC_1041, b) JC_1044, c) JC_0120 and d) control. Image pairs for each subject are left and right hemispheres. Rotating wedge stimuli were used to map the vertical and horizontal meridians corresponding to phase color reversals. White contours outline the hand delineated visual areas which corresponds to V3v, V2v, V1v, V1d, V2d and V3d respectively in the counter-clockwise direction.

A possible cause for the difference between our measurements and those of other investigators [6] could be that our visual stimulus had a fixation target to ensure that the subjects maintained their gaze at the center. This resulted in the absence of flashing checkerboard stimulation in this central area and may have caused a lack of coherence in the retinotopic data at the PRLF, thereby leading to a possible underestimation of the fovea. However, we feel that what is most relevant here is that we found no significant difference in foveal area between the BCM carriers and normal controls we measured. In addition, it is worth noting that out of the 4 BCM carriers, only one (JC_0120) had a slightly smaller foveal confluence (left = 844 mm^2^; right = 947 mm^2^) than the minimum of either control group (min of [6] = 982 mm^2^; min of controls = 1085 mm^2^), further demonstrating that the cortical size of the foveal confluence of the BCM carriers was within the normal range. The box plots in Figure 9 illustrate the similarity in the area of the foveal confluence and measurements of the entire surface area of V1, V2, and V3 for both the BCM carriers and our control group; results from Dougherty et al. [6] are shown for comparison. Measurements for each hemisphere were obtained by summing both the dorsal and ventral aspects. Figure 10 shows flattened pseudocolor phase maps for three BCM carriers and one control, with contours outlining the delineated foveal confluence. The area of V1/2/3 of the BCM carriers were not significantly different than the controls (p = 0.73; t-test, two sample; d = 0.1437). Phase maps showing the hand-delineated visual areas are shown in Figure 11.

## Discussion

### Contrast Sensitivity and Visual Acuity in BCM Carriers is Similar to Normal Observers

On psychophysical tests performed without AO, BCM carriers performed in the normal range. This confirms the findings of previous researchers, who showed that BCM carriers usually appear normal on most tests of visual function. This is in line with the large amount of variability in what is considered to be “normal” for the visual system. The contribution of this work is to establish such apparently normal vision given the significant amount of disruption observed at the level of the cone mosaic. Indeed AO-corrected measurements revealed a very different pattern of visual resolution, with BCM carriers having much poorer resolution than controls once optical aberrations are no longer a limiting factor. In fact, contrary to the relationship found in normal eyes, where optical aberrations typically limit visual resolution in the fovea, cone spacing can limit visual resolution at the fovea in some BCM carriers.

The relative role of optical quality and retinal sampling in shaping our visual capacities is illustrated by considering closely the two carriers who underwent visual resolution testing both with spectacle correction only and with AO-correction. Despite some differences in the stimulus used in each experiment (e.g. polychromatic with spectacle correction only in Experiment I (MAR_SC_) and monochromatic with AO-correction in Experiment II (MAR_AO_)), it is interesting to compare the results obtained for these two carriers (JC_1043 and JC_1041). At the PRLF, JC_1041 obtained MAR_SC_ of 0.75 arcmin and a nearly identical MAR_AO_ of 0.74 arcmin. At 2.5 degrees the results were also nearly identical, where JC_1041 obtained MAR_SC_ of 2.12 arcmin and MAR_AO_ of 2.09 arcmin. For this observer, it appears that high order ocular aberrations, which are minimized with AO, had little effect on visual performance. The other BCM carrier tested in both experiments, JC_1043, obtained quite different measurements in each experiment. At the PRLF, JC_1043 had a MAR_SC_ of 0.86 arcmin and MAR_AO_ of 0.72 arcmin. At the 2.5° location she obtained MAR_SC_ of 2.19 arcmin and a MAR_AO_ of 1.46 arcmin at a slightly greater eccentricity of 2.64°.

The first patient, JC_1041 was thus not ‘optically’ limited under normal viewing conditions (ie. for MAR_SC_ measurements), but rather was limited by retinal sampling in both, while the latter JC_1043 was ‘optically’ limited under normal viewing conditions, as she improved when optical aberrations were minimized with AO (ie. for MAR_AO_ measurements, as normal observers do). However, she failed to reach the performance levels we have observed previously in normal eyes [31], [40], [62]. This illustrates how differences in optical quality alone can impact visual resolution, obscuring any differences that may exist downstream in the visual system of different observers. Visual resolution tests with AO can reveal these small differences, as has been shown previously in myopia [40].

### Fixational Eye Movements are Abnormal in Some BCM Carriers

The average SD of fixation for the normal observers when looking at the stimulus was similar to that observed by others [59], [63]. The larger SD of fixation when both groups of observers looked at the fixation target relative to the stimulus was not surprising as target size, luminance and color (all of which were different for the fixation target relative to the stimulus) have been shown to influence fixation stability [63]. The relatively larger spread of fixation found in the BCM carriers is consistent with the abnormalities in eye movements previously observed in BCM carriers [27]. It should be noted that JC_1043 had a pattern of fixation that was within the normal range at all locations; she is the carrier who also had the best resolution, highest cone density (lowest N_c_), and presumably the least amount of cone loss. This is consistent with previous findings that showed that although fixational eye movements may be abnormal in some BCM carriers, they are not abnormal in all BCM carriers [27]. An alternative explanation for the fixational instability seen in the BCM carriers might be that they simply were less familiar with the procedure than our normal controls.

It is interesting to note that for test locations at the PRLF, MAR_AO_ was moderately correlated with the SD of fixation for the carriers (R^2^ = 0.70) but not for the normal observers (R^2^ = 0.31). However, the larger motion probably did not cause the resolution deficit in the carriers, as it has been shown that visual resolution is largely unaffected by retinal image motion [64]. In fact, the relative fixational instability seen in two of the three carriers is probably a result of their increased N_c_ (and lower MAR_AO_), consistent with the hypothesis of Steinman and colleagues, that one fixates accurately in order to see clearly, not because one sees clearly [65]. It is possible that the fixation control mechanism is relaxed in BCM carriers because the larger cone spacing tolerates a larger degree of image motion without interfering with vision. That is, the carriers are probably less stable than normal because they can tolerate a larger amount of retinal image motion without it interfering with their ability to see clearly.

### Does Cone Loss Lead to RGC Loss in the BCM Carrier Retina?

In normal observers, the Nyquist limit of the mosaic of midget retinal ganglion cells (N_mRGC_) predicts the reduction in visual resolution seen outside the foveola [31]; N_mRGC_ is set by the density of mRGC receptive fields. The transition from N_c_-limited visual resolution in the foveal center to N_mRGC_-limited resolution across the visual field is a consequence of eccentricity-dependent changes in retinal circuitry in the cone to midget bipolar cell to mRGC network [31], [61]. When the ratio of mRGCs to cones is 2 or greater, as it is expected to be in the center of the normal fovea [61], the expectation is that each cone will have a so-called ‘private line’ connection [66] to each of an ON- and OFF- centered mRGC. When this circuitry exists, the Nyquist limit of mRGC receptive fields is identical to N_c_ (i.e. N_mRGC_ = N_c_). Under these conditions MAR_AO_ is expected to match N_c_.

In the normal retina, the density of both mRGC receptive fields and cones decrease with eccentricity [1], [67], as does the mRGC-to-cone ratio [61]. When this ratio falls below 2, the centers of mRGC receptive fields begin to receive input from more than one cone. This compromises resolution, resulting in MAR_AO_ no longer matching N_c_ but rather matching N_mRGC_ [31]. If the reduced number of cones in the BCM carrier retina was paired with a normal number of RGCs, the BCM carrier retina would have a higher mRGC-to-cone ratio across the retina than is found in normal eyes. For example, if a BCM carrier fovea had half the cones of a normal retina but a normal complement of mRGCs, the mRGC-to-cone ratio might be 4∶1 instead of the 2∶1 ratio found in the normal retina. Because the mRGC-to-cone ratio governs the eccentricity at which the transition from N_c_ limited resolution to N_mRGC_ resolution occurs, it is expected that this transition would occur at a more eccentric location in the carrier retina than in the normal retina, extending the ‘private line’ connection to greater eccentricities. This hypothesis is summarized in supplementary figure S1, which shows the circuitry and acuity predictions made under this scenario. Under this scenario, N_c_ might be expected to match MAR_AO_ across the full range of test locations examined herein. However, we found that MAR_AO_ and N_c_ are matched only at the PRLF and that MAR_AO_ falls off at a greater rate than predicted by N_c_ just outside the PRLF, with a pattern that is similar to that observed previously for normal observers [31].

This result supports the hypothesis that there are postreceptoral changes in the organization of the BCM carrier retina. One possibility is that there is coordinated variation between the number of cones and the number of ganglion cells: the number of ganglion cells is reduced in BCM carriers in a systematic way. Support for this hypothesis comes from the fact that we show that the theoretical N_mRGC_ based upon mRGC receptive field density measurements from normal eyes [61] does not fit with the data obtained from BCM carriers, except for the most eccentric test location of JC_1043 (where she had normal cone density). BCM carrier performance at all other test locations was worse than predicted by the model of Drasdo et al. [61], providing further evidence that there is loss of mRGCs. That this loss is local, and is a consequence of the cone loss, is supported by the results from JC_1043 at the most eccentric test location. This BCM carrier was within the normal range on all measures at this test location, and fit well with the model prediction for N_mRGC_ limited resolution at that test location. This finding is consistent with coupled cone-RGC loss, as a reduction in mRGC density is predicted only where reduced cone density is observed.

### The Relationship between AO-corrected Visual Resolution (MAR_AO_) and the Nyquist Limit of the Cone Mosaic (N_c_) in BCM Carriers is Similar to that Observed for Normal Eyes Despite Evidence for Coupled Cone-mRGC Loss in the Carrier Retina

Examining in detail the relationship between N_c_ and MAR_AO_ found in the BCM carrier retina, it can be seen that the relationship observed is similar to what was observed in both the normal retina and to what is predicted from the model of Drasdo and colleagues [61]. As can be seen from Figure 6a, for a given N_c_, BCM carriers actually achieved better MAR_AO_ than the myopic control eye and fell well within the limits measured previously for normal eyes [31]. However, the eccentricities at which equivalent MAR_AO_–N_c_ pairings were found in the BCM carriers were much closer to the PRLF than in a normal retina. For a given MAR_AO_, the difference between N_c_ and MAR_AO_ is similar in both the normal and carrier retina. In fact, corresponding values of N_c_ and MAR_AO_ found in the carriers were similar to model predictions at all test locations, the only difference being that they were not found at the model-predicted retinal eccentricities.

**Figure 12:**
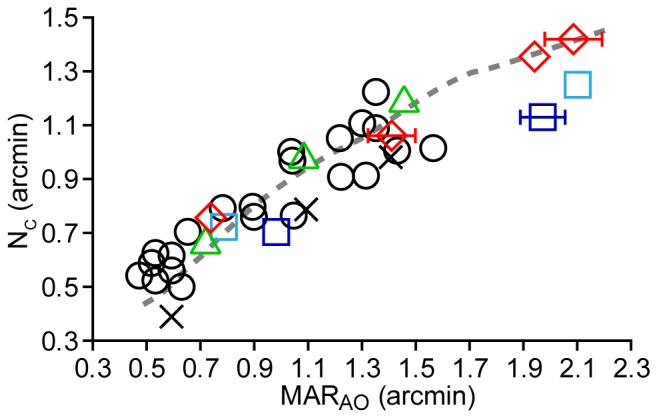
Relationship between MAR_AO_ and N_c_ in BCM carriers is similar to normal eyes. Data from figure 6a is shown with model predictions and data for normal eyes from Rossi & Roorda (2010). JC_1043, JC_1041, JC_1045 (OD), JC_1045 (OS) are shown as green triangles, red diamonds, dark blue squares, and light blue squares, respectively. The control eye is shown as a black X. Observers from Rossi & Roorda (2010) are shown as black circles. The dashed grey line shows the mean N_c_ of Curcio et al. (1990a) plotted against N_mRGC_ (an estimate of the neural MAR) from the model of Drasdo et al. (2007).

Figure 12 re-plots the data shown in Figure 6a, along with the data from the normal observers from Rossi & Roorda [31], and a curve showing the theoretical relationship between N_c_ and the N_mRGC_ predicted from the Drasdo et al. model [61] and the cone density data of Curcio et al. [1]. For each eccentricity at which an N_c_ measurement was calculated, a corresponding value of N_mRGC_ was computed using the general model of mRGC receptive field density of Drasdo and colleagues [61]. It can be seen that both the normal and BCM carrier data points fall near the model prediction curve. Points near the PRLF for the empirical data are slightly shifted upward from model predictions. This is not surprising as N_c_ was estimated at the PRLF for four of the five observers from Rossi and Roorda [31]; these estimates contain errors and are, on average, higher than N_c_ estimates derived from the peak density measurements of Curcio [1]. It is also likely that the estimates of N_c_ from the average data of Curcio contain some error because the true conversion factor (*m*) for those eyes between mm and degrees of visual angle is unknown. The large range of cone densities observed at the foveal center also makes predictions at this location subject to the largest amount of variability.

The agreement between model predictions and empirical data was better outside the PRLF, where measurements of N_c_ were made directly. It is interesting to note that the BCM carrier data points of MAR_AO_ values beyond the range of those obtained for normal observers still follow the trend predicted by the model. This shows that the carrier retina can be thought of, in effect, as an eccentricity-shifted version of the normal retina. For example, observer JC_1041 had a MAR_AO_ of 1.41 arcmin when her N_c_ was 1.06 arcmin, falling near the model prediction for that MAR_AO_–N_c_ pairing; however, the model predicts that this combination would be found at an eccentricity of ∼2.3° in a normal retina, whereas it was observed at an eccentricity of only 0.86° degrees in JC_1041, a difference of 1.44°.

What does this tell us about the retinal circuitry of the BCM carrier retina? It seems to indicate that for a given cone spacing, downstream neural circuitry is similar in both normal and BCM carrier retinas. This suggests that the main determining factor for the size of mRGC receptive fields appears to be the spacing between cone photoreceptors, as the relationship between N_c_ and MAR_AO_ appears to be identical in both groups. As such, this finding indicates that N_mRGC_ is largely determined by N_c_. This suggests that the number of mRGCs in the retina is directly related to the number of cones and that the cone loss seen in the carriers probably led to a subsequent loss of RGCs. It is hard to imagine how the visual system might deal with a mismatch between the number of cones and mRGCs, particularly in the case where there would be either redundant circuitry at the midget bipolar or midget ganglion cell level, which leads to such strange predictions as spatially redundant mRGC receptive fields in the center of the BCM carrier fovea (see supplementary figure S1). More plausible is the prediction that the ’private line’ might persist to larger eccentricities than found in the normal retina, well outside the PRLF. However, it is not clear how a cone-mRGC ratio of greater than 2∶1 (which is implied if a normal complement of mRGCs is paired with a reduced number of foveal cones) would be implemented at the foveal center or if it would be advantageous. The ON- and OFF- center mRGC sub-mosaics are thought to be spatially redundant in the central fovea, requiring 2 mRGCs per cone; it does not seem plausible for there to be multiply redundant ON and OFF arrays or that there is a mechanism for such circuitry to be implemented. There may be a spatial limit to the size of a single-cone centered mRGC receptive field, as a discord between MAR_AO_ and N_c_ at the PRLF in the carrier eye with the largest cone spacing was observed. However, as a discord between MAR_AO_ and N_c_ at the PRLF was also observed for the control eye, this question cannot be answered here, and it is likely that cortical factors are also involved in limiting MAR_AO_ in the BCM carrier at the foveal center, as is probably the case in myopia [40]. The primary limitation of this study is that we did not obtain fMRI and mRGC estimates in all subjects, precluding direct comparison between the cortical representation of the fovea and our predictions of RGC densities in the same subjects. However, the results from the group of BCM carriers that we did measure clearly suggest that the cortical retinotopic organization in BCM carriers is no different from that seen in normal eyes. Further understanding of the organization of RGC receptive fields near the PRLF and the relationship between mRGC density and the cortical representation is required.

What emerges from the results of this set of experiments is perhaps a simple explanation about what governs the foveal overrepresentation in the cortex (ie. cortical magnification). It is well known that in normal eyes, cones greatly oversample the retinal image, but there are other constraints on the information available to the cortex than those specified by the number of cones. Despite reduced cone density, all BCM carriers appeared within the normal range on conventional tests of visual function: this is perhaps the key finding here. This suggests that it is not the number of afferents that drives the foveal overrepresentation, but rather the content of the information that is relayed to the cortex from the retina across the visual field. Under normal viewing conditions, filtering of information limited performance at all locations in the visual field for all observers. Although the filtering mechanisms differed for BCM carriers and normal observers, the resulting information reaching the cortex under normal viewing conditions was similar. At the PRLF, the filtering mechanism was the optics of the eye for the normal observers but cone spacing for most of the BCM carriers. Outside the PRLF, optical filtering probably continued to limit performance in normal eyes very close to the PRLF, but at the more eccentric locations, neural filtering (ie. convergence) imposed the limit in both groups. The filtering mechanism is invisible to the cortex; the information it receives is comparable in each case. In both groups, the central visual field was the most finely sampled area and thus contained the most information about each unit of visual space. The cortical area represented by the fovea and periphery was similar in both groups, suggesting that the information processing requirements were much the same.

This study demonstrates how genetic, behavioral, and imaging techniques can be applied in concert to develop a more comprehensive understanding of the interdependence between different structures within the visual system. This approach is challenging in that it requires technical expertise that few, if any, individual laboratories possess and so requires collaboration amongst several investigators. Despite these challenges, this approach may be well suited to study other conditions that disrupt the organization of the interrelated components of the visual system, such as inherited retinal diseases.

### Conclusions

1. Visual acuity and contrast sensitivity testing without AO reveals no differences between BCM carriers and normal observers.
2. AO-corrected visual acuity testing reveals that AO-corrected visual resolution is worse in BCM carriers than in normal eyes.
3. Fixational eye movements are abnormal in the BCM carriers with the most cone loss.
4. The relationship between AO-corrected visual resolution and the Nyquist limit of the cone mosaic is similar to normal in BCM carriers, suggesting that cone spacing largely governs the spacing of midget ganglion cell receptive fields.
5. Cone loss in BCM carriers leads to loss of mRGCs in the retina, suggesting that cone density governs ganglion cell density in the human retina.
6. Retinotopic mapping showed that despite the loss of cones, and evidence supporting a loss of mRGCs, the foveal overrepresentation in the cortex (ie. cortical magnification) is indistinguishable from normal in BCM carriers.

Taken together, these results suggest that ganglion cell density may not govern the foveal overrepresentation in the cortex and that it is perhaps driven solely by the information it receives from the retina.

## Supporting Information

Figure S1 **Predicted retinal circuitry, mRGC-to-cone ratio, and resolution, based on a model where cones are lost in BCM carriers but not mRGCs.** Left panel shows normal retinal circuitry; right panel (shaded) shows predictions for a BCM carrier retina where half of the cones are lost. a) Model predictions for the center of the fovea. At the foveal center, the normal eye has a mRGC-to-cone ratio of 2∶1; at this location MAR_AO_ matches N_c_, which also matches N_mRGC_. For the BCM carrier, the predicted mRGC-to-cone ratio is 4∶1, there is redundant circuitry at the midget bipolar cell level, and there are pairs of mRGCs with spatially overlapping receptive fields; the resolution prediction is the same as in the normal retina. b) Model predictions for a location outside the center of the fovea. At this eccentric location, the mRGC-to-cone ratio has fallen to 1∶2 in the normal eye, MAR_AO_ is now equal to N_mRGC_; this limit is imposed by convergence of 2 cones onto a single mRGC. For the BCM carrier, cone loss results in redundant circuitry at both the midget bipolar and mRGC level. The mRGC-to-cone ratio is 2∶1, identical to that seen in the center of the fovea in normal eyes, allowing ‘private line’ circuitry to persist and predicting that MAR_AO_ still matches N_c_.
(TIF)
