# Supplementary figures and images for: Visual Function and Cortical Organization in Carriers of Blue Cone Monochromacy
Source: PLoS One. 2013 Feb 28;8(2):e57956. doi: 10.1371/journal.pone.0057956 (PMC3585243; doi:10.1371/journal.pone.0057956)

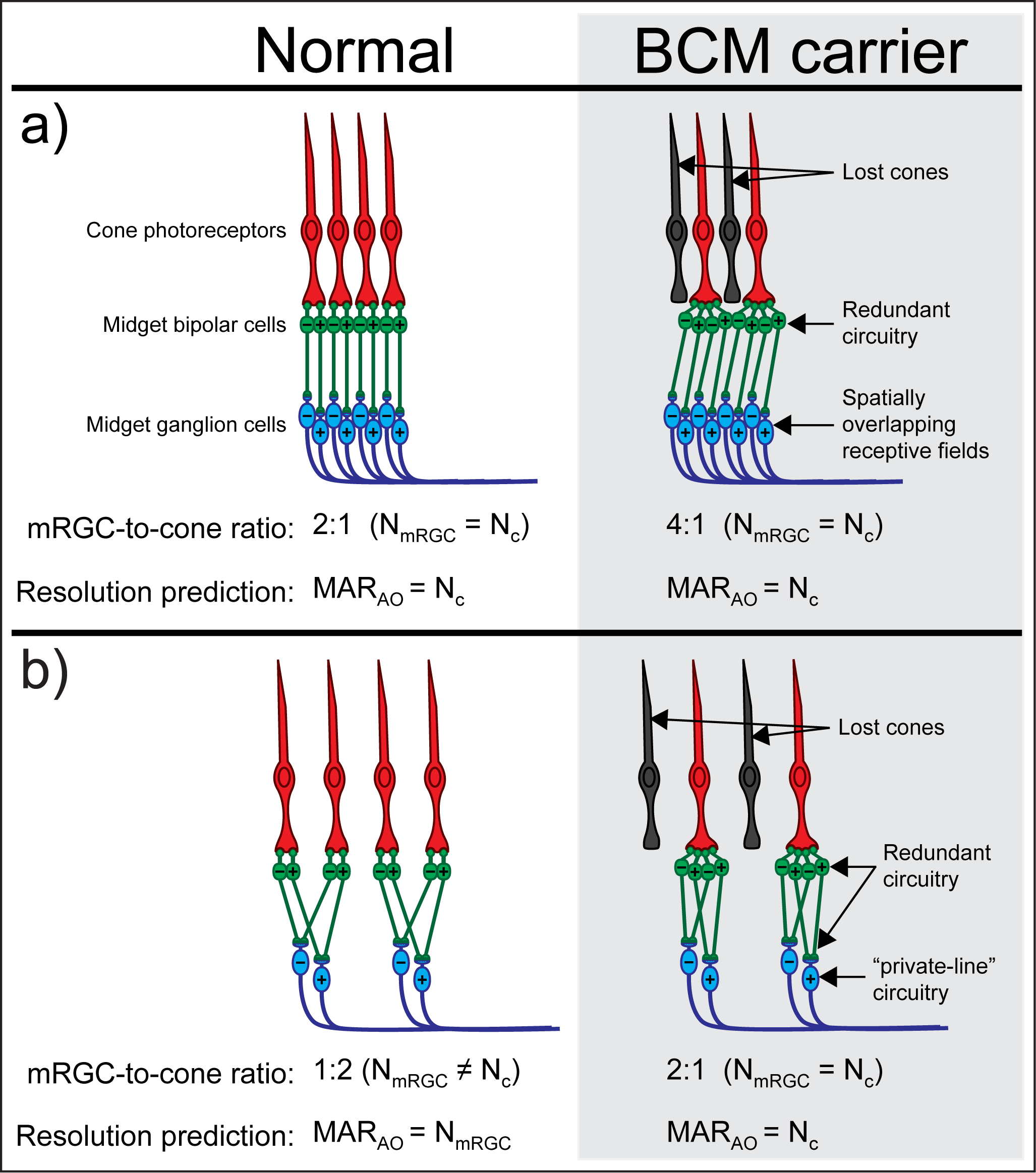

Supplement: Figure S1 — Predicted retinal circuitry, mRGC-to-cone ratio, and resolution, based on a model where cones are lost in BCM carriers but not mRGCs. Left panel shows normal retinal circuitry; right panel (shaded) shows predictions for a BCM carrier retina where half of the cones are lost. a) Model predictions for the center of the fovea. At the foveal center, the normal eye has a mRGC-to-cone ratio of 2∶1; at this location MARAO matches Nc, which also matches NmRGC. For the BCM carrier, the predicted mRGC-to-cone ratio is 4∶1, there is redundant circuitry at the midget bipolar cell level, and there are pairs of mRGCs with spatially overlapping receptive fields; the resolution prediction is the same as in the normal retina. b) Model predictions for a location outside the center of the fovea. At this eccentric location, the mRGC-to-cone ratio has fallen to 1∶2 in the normal eye, MARAO is now equal to NmRGC; this limit is imposed by convergence of 2 cones onto a single mRGC. For the BCM carrier, cone loss results in redundant circuitry at both the midget bipolar and mRGC level. The mRGC-to-cone ratio is 2∶1, identical to that seen in the center of the fovea in normal eyes, allowing ‘private line’ circuitry to persist and predicting that MARAO still matches Nc. (TIF) [file pone.0057956.s001.tif]
